# Multidisciplinary insight into clonal expansion of HTLV-1–infected cells in adult T-cell leukemia via modeling by deterministic finite automata coupled with high-throughput sequencing

**DOI:** 10.1186/s12920-016-0241-2

**Published:** 2017-01-31

**Authors:** Amir Farmanbar, Sanaz Firouzi, Sung-Joon Park, Kenta Nakai, Kaoru Uchimaru, Toshiki Watanabe

**Affiliations:** 1 Department of Computational Biology and Medical Sciences, Graduate School of Frontier Sciences, The University of Tokyo, Tokyo, Japan; 2 Laboratory of Functional Analysis in silico, Human Genome Center, Institute of Medical Science, The University of Tokyo, Tokyo, Japan; 3 Hematology/Oncology, Research Hospital, Institute of Medical Sciences, The University of Tokyo, Tokyo, Japan; 4 Department of Advanced Medical Innovation, St. Marianna University School of Medicine, Kanagawa, Japan

**Keywords:** Mathematical computational modeling, Deterministic finite state automata (DFA), State-transition diagram, Adult T-cell leukemia, Human T-cell leukemia virus ype-1, Integration site, Clonal expansion, Next-generation sequencing

## Abstract

**Background:**

Clonal expansion of leukemic cells leads to onset of adult T-cell leukemia (ATL), an aggressive lymphoid malignancy with a very poor prognosis. Infection with human T-cell leukemia virus type-1 (HTLV-1) is the direct cause of ATL onset, and integration of HTLV-1 into the human genome is essential for clonal expansion of leukemic cells. Therefore, monitoring clonal expansion of HTLV-1–infected cells via isolation of integration sites assists in analyzing infected individuals from early infection to the final stage of ATL development. However, because of the complex nature of clonal expansion, the underlying mechanisms have yet to be clarified. Combining computational/mathematical modeling with experimental and clinical data of integration site–based clonality analysis derived from next generation sequencing technologies provides an appropriate strategy to achieve a better understanding of ATL development.

**Methods:**

As a comprehensively interdisciplinary project, this study combined three main aspects: wet laboratory experiments, in silico analysis and empirical modeling.

**Results:**

We analyzed clinical samples from HTLV-1–infected individuals with a broad range of proviral loads using a high-throughput methodology that enables isolation of HTLV-1 integration sites and accurate measurement of the size of infected clones. We categorized clones into four size groups, “very small”, “small”, “big”, and “very big”, based on the patterns of clonal growth and observed clone sizes. We propose an empirical formal model based on deterministic finite state automata (DFA) analysis of real clinical samples to illustrate patterns of clonal expansion.

**Conclusions:**

Through the developed model, we have translated biological data of clonal expansion into the formal language of mathematics and represented the observed clonality data with DFA. Our data suggest that combining experimental data (absolute size of clones) with DFA can describe the clonality status of patients. This kind of modeling provides a basic understanding as well as a unique perspective for clarifying the mechanisms of clonal expansion in ATL.

**Electronic supplementary material:**

The online version of this article (doi:10.1186/s12920-016-0241-2) contains supplementary material, which is available to authorized users.

## Background

Cancer is a complex disease of the genome that behaves as a clonal evolutionary process in populations of cells [1–4]. Although cancer is a diverse and multifactorial disorder with differing origins and degrees of malignancy, clonal expansion and the presence of Darwinian or natural selection are generally accepted as common features [4, 5]. Since Nowell first proposed the clonal evolution theory of neoplasia in 1976 [1], a broad range of studies have provided support for this model. In recent years, the use of next-generation sequencing (NGS) technologies for the investigation of tumor genomes has generated increasing evidence that most neoplasms grow as a clonally expanded cell population [3, 6–8]. The vast amounts of invaluable data generated by NGS have surpassed analysis and interpretation capacity. However, the intricate nature of clonal expansion and evolution in cancer makes it difficult to convert the experimental and clinical data into medical practices [9, 10]. Experimental data alone are not generally sufficient enough to address the complex problem of cancer. Consequently, focus has shifted toward devising mathematical/computational models for simplification and extraction of fundamental meaning from the complex biological processes of cancer.

Adult T-cell leukemia (ATL) is a life-threatening malignancy that manifests with very poor prognosis [11, 12]. ATL develops through a multistep leukemogenic process, the nature of which remains elusive [13]. Among the different types of cancer, ATL is a remarkably unique neoplasm in that it is directly caused by infection with human T-cell leukemia virus type-1 (HTLV-1), which is mainly transmitted via breastfeeding [14]. HTLV-1 infection and integration of provirus into the host genome are intrinsic and inevitable early events for ATL development [15]. HTLV-1 mainly survives *in vivo* by persistent clonal proliferation of infected cells [16]. Whereas the majority of HTLV-1–infected individuals remain asymptomatic carriers (ACs) throughout their lifetime, ~5% of them develop ATL after a long period of clinical latency [17]. Currently, there is no clear determinant to distinguish between individuals who will remain ACs and those who will develop ATL [18, 19]. Our Joint Study on Predisposing Factors of ATL Development (JSPFAD) group examined ATL risk factors and demonstrated that a proviral load (PVL; i.e., the percentage of infected peripheral blood mononuclear cells) of >4% is one of the risk factors for progression to ATL; however, PVL alone cannot predict development of the disease [19]. Similar to other types of cancer, clonal expansion of abnormal cells is a hallmark of ATL [20, 21]. Considering that the incidence of large clones increases with disease progression from the healthy AC state to the malignant states of smoldering (SM), chronic, or acute ATL [22–24], monitoring clonal expansion via an accurate method of detection is of great clinical importance [8, 23].

Generally, mutation patterns of cells can be used to define clones and monitor clonal expansion in different types of cancer [7]. ATL development has an advantage in that not only the mutation pattern but also the integration site of the provirus can be used to define clones and monitor clonal expansion [8, 23]. Individual infected cells can be uniquely characterized based on their integration site because, typically, a single integration of HTLV-1 occurs per host cell [25]. Detecting the clonality dynamics, including clonal status and alterations, requires an appropriate method for defining two main characteristics of clones, HTLV-1 integration site and clone size.

Research in this area would be greatly benefitted by an easier to understanding representation and description of how cancer develops in terms of clonal expansion, which is expected to be provided by appropriate models. A realistic model would provide a better understanding of cancer and would provide a comprehensive perspective on cancer processes by integrating clinical and biological data within a mathematical and computer science framework. As with other malignancies, suitable models for ATL would help to simplify the dynamics of cooperative and complex behaviors in cancer development [26, 27]. Quantitative NGS data have the potential for creating robust and reliable mathematical modeling approaches [28]. Increasingly complex mathematical models of cancerous growth (particularly of solid tumors) that are based primarily on mutation patterns have been developed [29, 30]. The prominent role of mathematical modeling in the detailed quantitative description of diseases, and the contribution of mathematical modeling to solving biological problems have been eloquently discussed by Tanaka and Ono [31]. Currently, there is a broad range of theoretical models available; however, empirical mathematical models are still limited [30].

Several mathematical modeling studies are available in the field of HTLV-1 research [32–38], although none of these studies have focused on modeling clonal expansion and its correlation with ATL development. The earliest mathematical model for HTLV-1 explored the correlation between the antiviral immune response, viral load and viral diversity [32]. Later Stilianakis et al. used a nonlinear differential equation and theoretical assumptions to describe HTLV-1 infection of CD4^+^ T-cells [34]. This model was further optimized to test different assumptions and/or alteration of the proposed differential equations [35–38]. Therefore, there is an obvious lack of a data-driven mathematical model that describes the role of clonal expansion of HTLV-1–infected cells in ATL development. Mathematical models that are data-driven and hypothesis-free are considered to be the most applicable in many situations and have the lowest risk of confirmation bias [31]. Moreover, there is currently no computational model available for ATL development. A model that not only reflects details of biological phenomena like mathematical models but also allows abstract visualization of the observed information like computational models would be most informative to biologists [39]. Establishing suitable expressive formalisms requires filling the gap between mathematics and computer science by using advantages of both approaches.

In this study, we used deterministic finite state automata (DFA), which are a concept in automata theory [40]. Automata are the main mathematical objects in computer science that are capable of applying sequential algorithms, formalism, to system description and specification [41, 42]. DFA can abstractly display evolutionary processes and other phenomena with a sequential order of events [40, 43]. DFA represent a framework to describe the behavior of clonal expansion as discrete-state systems. Our main goal was to illustrate clonality patterns and to design a conceptually clear framework based on real biological data on clonality obtained from individuals with different PVLs and progression states of ATL. We also categorized the observed clone sizes accurately based on our integration site–mediated clonality analysis approach. Moreover, we propose the first well-suited empirical model for intuitive description of clonal expansion in ATL.

## Methods

### Wet laboratory experiments

HTLV-1–infected individuals harbor complex populations of infected clones and uninfected cells [8, 23]. HTLV-1 integration sites and the number of infected cells in each clone (i.e., clone size) are two main characteristics of infected clones that we monitored. Each HTLV-1–infected cell naturally harbors only a single integration site [25]. Therefore, the number of detected unique integration sites reflects the number of infected clones. The most challenging aspect of our clonality analysis was measuring the number of infected cells in each clone. We used a molecular tagging system for this purpose. Tags acts as molecular barcodes which give DNA fragment unique signatures before PCR [8]. Information on the frequency of observed tags from the NGS data was used to remove PCR duplicates and thereby estimate the original clonal abundance in the starting sample. Because of the random design of tags, they could theoretically provide ~65,536 variations, and thus can uniquely mark a large number of cells in each clone. This method has been comprehensively validated using control samples with known clone sizes and clinical samples [8].

In total, eighteen clinical samples were obtained from the JSPFAD biomaterials bank of HTLV-1 carriers [44, 45]. Samples Information is provided in Table 1. The clinical samples were collected with written informed consent as a collaborative project of JSPFAD. The project was approved by research ethics committee of the University of Tokyo. Information about the disease status of samples was obtained from the JSPFAD database in which HTLV-1–infected individuals were diagnosed based on the Shimoyama criteria [46].

**Table 1:** Sample characteristics

Sample	Clinical status	PVL (%)	DFA machine	Final state	Integration sites
F1	AC	7.57	M1	q1	876
F2	AC	5.24	M1	q1	802
F3	AC	7.16	M1	q1	1473
F4	SM	6.02	M1	q1	1827
F5	SM	31.15	M4	q2	225
F6	SM	23.56	M2	q2	398
F7	SM	36.63	M4	q2	570
F8	SM	43.24	M7	q3	417
F9	Chronic	28.53	M4	q2	260
F10	Chronic	15.25	M4	q2	1345
F11	Chronic	100.70	M3	q3	73
F12	Chronic	83.81	M3	q3	65
F13	Acute	64.43	M6	q3	138
F14	Acute	27.92	M5	q3	390
F15	Acute	51.90	M3	q3	40
F16	Acute	51.42	M3	q3	19
F17	AC	1.24	ND	ND	233
F18	AC	3.52	ND	ND	739

*DFA* deterministic finite state automata, *PVL* proviral load, *AC* asymptomatic carrier; *SM* smoldering, *ND* not determined

To prepare the samples for sequencing, 5 μg genomic DNA from peripheral blood mononuclear cells was isolated using a QIAGEN DNA Blood kit. PVLs were measured by real-time PCR using the ABI PRISM 7000 Sequence Detection System as described [19].

We used a library preparation protocol specifically designed to isolate HTLV-1 integration sites. All information about the design and detailed protocols has been described [8]. In brief, the starting template DNA was fragmented by sonication. The resulting fragments represented a size range of 300 to 700 bp as indicated by an Agilent 2100 Bioanalyzer and DNA 7500 kit. Fragmented DNA underwent the library construction steps of end repair, A-tailing, adaptor ligation, size selection and nested PCR. The generated products contained all the specific sequences necessary for the Illumina HiSeq 2000/2500 platform (Additional file 1: Figure S1).

### *In silico* analysis

We analyzed the large amount of NGS data with a pipeline specifically designed for HTLV-1 integration sites and clone size measurement. We processed raw sequencing data according to the workflow that we previously reported [8]. Briefly, raw data of Read-1 (100 bp forward), Read-3 (100 bp reverse), and Read-2 (8 bp index) were obtained from the Illumina HiSeq 2000/2500 platform. The quality of sequencing outputs was confirmed with the FastQC tool [47]. In the case of Read-1, the first 5 bp were trimmed, and the next 5 bp were used to de-multiplex indexed samples. The following 23 bp, which correspond to the long terminal repeat primer, were then removed. The next 27 bp were subjected to a BLAST search [48] against the long terminal repeat reference sequence. For the BLAST output reads, the remaining 40 bp were subjected to a BLAST search against an HTLV-1 reference sequence [49]. Reads confirmed to be from HTLV-1 were removed, and the sequences and IDs for the remaining reads, which were considered to be human, were collected. Subsequently, reads from Read-3 with IDs corresponding to IDs from Read-1 were collected. The first 40 bp of Read-3 were trimmed to have the same length as Read-1 sequences. The paired sequences of Read-1 and Read-3 were mapped against the human genome (version 19) by Bowtie [50]. For each sample, two million mapped reads were used for subsequent analysis. The 5′-mapped positions were considered to be integration sites. The output format of isolated integration sites is chromosome:position (strand) (e.g., chr7:9408533 (−)). Subsequently, Read-2 information, which contained 8-bp randomly designed barcodes, was used to retrieve the clone size based on the tags. Finally, clone size was measured by computing the frequency of unique tags per each integration site.

### Expressing results via empirical modeling

Formal definition can precisely describe automata by alphabet and formation rules in mathematics. Parameters of DFA, such as the number of accept states and the number of transitions exiting from a state, can be clearly defined by the formal definition. In mathematical language, a DFA is a 5-tuple where the components are (Q, Σ, δ, q0, F). “Q” is a finite set of states. Σ is a non-empty finite set of symbols (inputs). Transition rules are denoted by a function called the transition function, δ: States × Alphabet → States (δ: Q × Σ → Q). “q0” is a start state, where q0 ∈ Q. “F” indicates final states that are a subset of states Q [40, 42, 51].

## Results

### Analyzing clonality of clinical samples by high-throughput sequencing

**Fig. 1:**
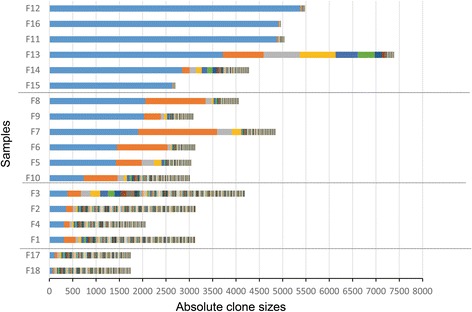
Clonality of samples with various PVLs. The clonal distribution in genomic DNA samples of the analyzed individuals. Each colored segment of a bar represents one unique integration site; the width of the segment is the clone size. Bars with segments of relatively similar sizes are considered to have relatively uniform distribution. The samples are displayed in ascending order based on the size of their largest clones

Having access to the biomaterials bank of JSPFAD [44, 45], we obtained 18 samples from HTLV-1–infected individuals with PVLs ranging from 1.24 to 100.7%. Detailed information on these samples is presented in Table 1. The results of our clonality analysis are presented in Fig. 1, and detailed information on the integration sites and clone sizes are provided in Additional File 1: Table S1. Samples F18, F17, F1, F2, F3 and F4 showed a uniform distribution pattern of clones with no large difference in clone size (polyclonal pattern). The size of the largest clone in each of these samples was 77, 112, 310, 357, 388 and 314 cells, respectively, and the PVLs were 3.52, 1.24, 7.57, 5.24, 7.16 and 6.02%, respectively (Fig. 1). F18 and F17 had PVLs lower than 4% and very small clone sizes. Samples F5, F6, F7, F8, F9 and F10 had non-uniform sizes (oligoclonal pattern). The size of the largest clone in each of these samples was 1427, 1446, 1904, 2055, 2029 and 736 cells, respectively; the size of the second-largest clone was 552, 1088, 1690, 1293, 361 and 725 cells; and the PVLs were 31.15, 23.56, 36.63, 43.24, 28.53 and 15.25% (Fig. 1, Table 1 and 2). Samples F11, F12, F13, F14, F15 and F16 harbored a dominant expanded clone (monoclonal pattern) with a high absolute number of infected cells. The largest clone size for each of these samples was 4883, 5377, 3721, 2848, 2634 and 4909 cells, respectively, and the PVLs were 100.7, 83.81, 64.43, 27.92, 51.9 and 51.42%, respectively (Fig. 1, Table 1 and 2). The PVL for each sample is also shown in the same order in Additional file 1: Figure S2. The PVLs of the samples and the sizes of the largest clone had a correlation of R^2^ = 0.785.

**Table 2:** The clone size and category of clones among Top-5 largest clones across all samples

F1		F2		F3		F4		F5		F6		F7		F8		F9		F10		F11		F12		F13		F14		F15		F16		F17		F18	
310	S	357	S	388	S	314	S	1427	B	1446	B	1904	B	2055	VB	2029	B	736	B	4883	VB	5377	VB	3721	VB	2848	VB	2634	VB	4909	VB	112	VS	77	VS
252	S	147	S	287	S	118	VS	552	B	1088	B	1690	B	1293	B	361	S	725	B	25	VS	14	VS	871	B	158	S	13	VS	17	VS	54	VS	29	VS
65	VS	58	VS	206	S	50	VS	263	S	67	VS	317	S	47	VS	69	VS	131	S	24	VS	8	VS	774	B	138	S	9	VS	3	VS	47	VS	28	VS
58	VS	43	VS	206	S	38	VS	161	S	24	VS	205	S	46	VS	64	VS	70	VS	12	VS	7	VS	769	B	121	VS	4	VS	2	VS	42	VS	27	VS
55	VS	35	VS	167	S	35	VS	60	VS	15	VS	20	VS	30	VS	52	VS	56	VS	11	VS	3	VS	475	S	117	VS	3	VS	2	VS	39	VS	26	VS

### Defining appropriate thresholds for the absolute clone size

**Fig. 2:**
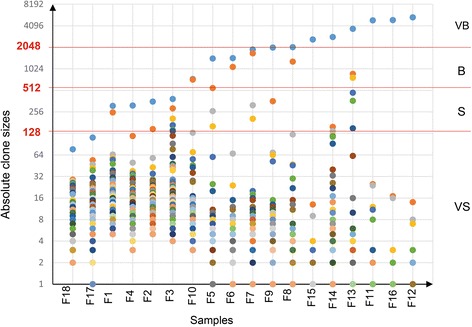
Distribution of clone sizes among the analyzed samples. Observed clone sizes were scatter plotted for each sample. The clone sizes are shown on a logarithmic scale. The red lines indicate the three thresholds of 128, 512 and 2048 cells distinguishing the four size groups, VS, S, B and VB

Each clone contains infected cells with identical integration sites. Based on the distribution of clone sizes (absolute number of infected cells in each clone) determined for the AC and ATL samples, we defined three thresholds for categorizing the clones (Fig. 2). In the simplest assessment, a cell with replication capacity N is theoretically capable of generating a colony of 2^N^ cells [21]. The clone sizes were densely distributed in the lower end of the size range and sparsely distributed in the upper end of the size range. To illustrate the pattern of clone size distribution, we show the distribution of the top five largest clones in Additional file 1: Figure S3. Based on the density distribution, we tested different thresholds and selected the thresholds (2^7^, 2^9^, and 2^11^) that best categorized clones. Using these three thresholds, we divided the observed clones into four distinct size groups: very small (VS, 1–128 infected cells), small (S, 128–512 infected cells), big (B, 512–2048 infected cells) and very big (VB, >2048 infected cells). The size and category of the top five clones across all samples are provided in Table 2.

### Taking advantage of automata theory to describe clonality data

**Fig. 3:**
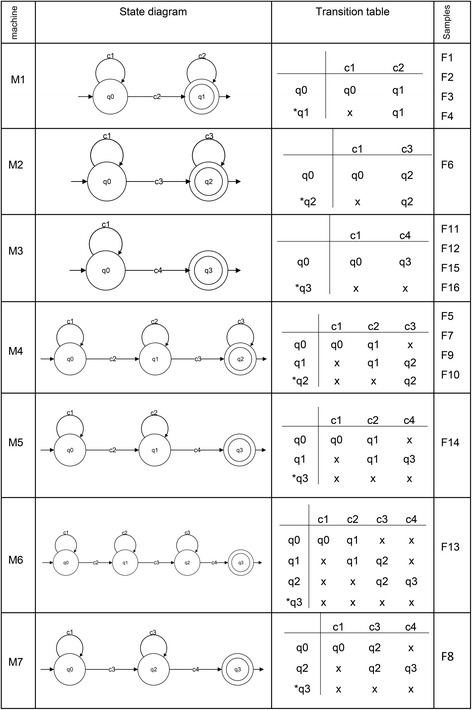
DFA machines for each sample. State diagrams and transition tables of the samples are represented by seven DFA machines (M1–M7). Asterisks indicate final states

**Fig. 4:**
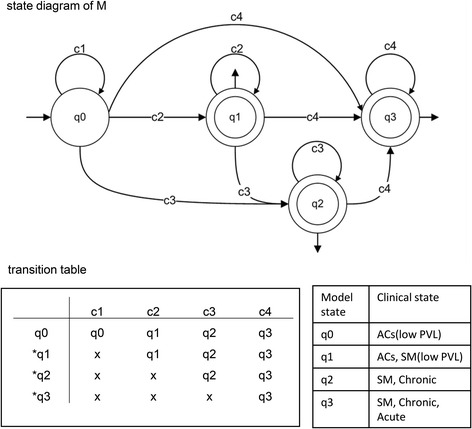
The main DFA machine representing clonality across all samples. Both the state diagram and transition table for all samples for machine M are shown. q0 is the start state; c1, c2, c3, and c4 correspond to VS, S, B, and VB, respectively. Asterisks indicate final states

The DFA in this study can present the observed clones in an ordered string based on their sizes, which is then accepted as input and then provide a graphical output that describes the clonality dynamics. In DFA, states and transitions can be graphically represented by circles and arrows, respectively. We defined the four states q0, q1, q2 and q3 for the machines. To start the analysis, the NGS-derived, quantified clone-size data were sorted in ascending order. Then, based on the threshold criteria that we described above, the sorted data were allocated to the VS, S, B and VB groups (Table 2), which were represented by the symbols c1, c2, c3 and c4, respectively. Our DFA read the clone size data as a finite string of symbols as an input. The set of all inputs is denoted by Σ* and can be any combination of c1, c2, c3, and c4. As an automaton sees an input, it decides whether to transition from one of a sequence of states (in our case denoted q0, q1, q2, q3, where qi ∈ Q, 0 ≤ i ≤ 3) to another. The transition function δ takes the current state and the recent symbol as its inputs. Fig. 3 illustrates the DFA machines for clonality data obtained from HTLV-1–infected individuals. According to the clonality data obtained from the clinical samples (Fig. 1 and Table 2), we designed seven DFA machines (M1–M7). To describe them informally, these machines are composed of different combinations of VS, S, B, and VB: [M1: VS, S], [M2: VS, B], [M3: VS, VB], [M4: VS, S, B], [M5: VS, S, VB], [M6: VS, S, B, VB], and [M7: VS, B, VB]. We represented these machines by state diagrams and transition tables in Fig. 3. The clonality patterns of F1, F2, F3 and F4 are modeled by M1; the pattern of F6 is modeled by M2; the patterns of F11, F12 and F15 are modeled by M3; the patterns of F5, F7, F9 and F10 are modeled by M4; the pattern of F14 is modeled by M5; the pattern of F13 is modeled by M6; and the pattern of F8 is modeled M7 (Fig. 3). Finally, to achieve a model describing all clonality data, we combined these seven machines and proposed our main machine (M) (Fig. 4). State q1 means that the clonality pattern is polyclonal, and the patient status is either AC or SM with low PVL. In other words, q1 accepts any combination of c1 and c2 clone sizes. State q2 means that the clonality pattern is oligoclonal, and the patient status is either SM or chronic, indicating that q2 accepts any combination of c1, c2 and c3 clone sizes. State q3 means that the clonality pattern is monoclonal or largely expanded oligoclonal, and the patient status is either SM, chronic or acute. In other words, q3 accepts any combination of c1, c2, c3 and c4. Our final DFA (M; Fig. 4) completely represents clonal expansion based on integration sites across all samples. DFA of AC and SM samples with low PVLs (F1, F2, F3 and F4) terminated in the final state of q1. DFA of SM and chronic samples (F6, F5, F7, F9 and F10) terminated in the final state of q2. DFA of SM, chronic and acute samples (F8, F11, F12, F13, F14, F15 and F16) terminated at q3.

## Discussion

Modern medicine has done much to eradicate and cure disease, but it has been less successful in some areas, such as cancer, which still remains one of the most common incurable diseases. Remarkable progress has been made recently in the genomics of cancer with the advent of NGS technologies. However, this explosion in rapidly generated, massive sets of loosely structured raw data has challenged our abilities to quantitatively analyze and draw knowledge from this information [52]. Formal modeling can address this problem by enabling appropriate simplification of real data and making sense of observed experimental data. Empirical modeling provides an accurate and complete picture of observed complex data, and it has many applications in the life science [53]. The merging of mathematics, computer science and biology in empirical models can reshape these fields by providing new ways of thinking about a problem. The virtue of mathematics in modeling is to confer clarity and precision to explanations, and to provide coherence and formalism to experimental observations [54]. The quantitative and objective power of mathematics allows understanding of otherwise hidden aspects of biological phenomena [28]. Computational models enable intuitive representation of the masses of biological data via their visualization capability, which in turn facilitates mechanistic understanding of disease [55].

The most effective and appropriate type of mathematical/computational modeling varies for each biological question. A practical model that is properly formulated to explain and interpret experimental and clinical data obtained from analyzing clonal expansion in ATL is greatly needed. We need a model that can imitate the components of our biological system (the clonality patterns and clone sizes) and reflect its properties intuitively.

In the current study, we aimed to organize and intuitively express data from NGS on clonal expansion of HTLV-1–infected individuals using finite automata theory. Finite automata theory can describe and analyze dynamic behaviors of systems, and it is capable of simply representing complicated processes [43, 52]. Finite automata theory, which is a well-developed formal system, is used in processing various strings and sequences, especially in DNA sequence processing [51]. DFA are a subtype of finite automata theory and are simple computational structures that can formally illustrate the size order and combination of observed clones (clonality patterns). Our model translates the observed data into formal mathematical language by formulating a precise relationship between a set of clones in terms of their sizes and presenting this relationship in an easily understood state diagram.

Conventionally, clonality has been described as polyclonal, oligoclonal and monoclonal [56, 57]. However, these pattern descriptions are not quantifiable. For instance, it is known that the monoclonal expansion that results in large clones is an intrinsic feature of ATL development [20]; however, absolute clone sizes to describe this phenomenon have not been determined. In recognition of this limitation, we categorized the observed clone sizes into defined groups by which we could intuitively assess the degree of clonal expansion. We defined four groups of clonality patterns and four groups of clone sizes. Thus we defined polyclonal as a pattern showing different combinations and large numbers of VS and/or S clones, oligoclonal as a pattern showing more than one B or VB clone in combination with large numbers of VS and/or S clones, and monoclonal as a pattern showing a single VB or B clone in combination with a background of VS and/or S clones. In this way, we could attribute a meaning to the observed clone sizes and assess their contribution to ATL progression. In other words, we quantified how large a clone must be to affect the clinical status of an infected individual.

Generally, it is known that competition between clones shapes their distribution [3], but we do not know how a clone wins this competition to undergo clonal expansion. Presumably, a clone needs to become large enough to gain a fitness advantage to out-compete other clones. Coexistence of large numbers of S and VS clones, as well as presence of limited numbers of B or VB clones together with large numbers of S or VS clones in each given sample was observed. Total number and type of isolated clones are provided in Table 1, and Table 2. Therefore, we suggest that small cell populations (VS and S) do not have a selective advantage and can coexist with other clones. ACs and patients with SM ATL with low PVL harbored only VS and S clones, whereas all dominant clones in aggressive ATL (acute) were VB. The observed clone sizes were sorted in ascending order, and then thresholds of 2^7^, 2^9^ and 2^11^ cells were applied. Hence, observed clone sizes were categorized into four distinct groups. Over the threshold of 2^11^ cells, the largest clones in the samples that had monoclonal patterns were categorized within the same group. Within the threshold range of 2^9^ to 2^11^ cells, the two largest clones in the samples that had oligoclonal patterns were categorized in the same group. Within the threshold range of 2^7^ to 2^9^ cells, clones in the samples that had polyclonal patterns with PVLs > 4% were categorized in the same group. Below the threshold of 2^7^ cells, the clones in the samples that had polyclonal patterns with PVLs < 4% were categorized in the same group. This quantified categorization of clone size not only is more intuitive for biological interpretation but also facilitates the transferal of clone size information into our model.

To convert the complex nature of data on clonal expansion into a manageable level of simplicity, borrowed the aid of mathematics and computer science. Our proposed DFA describe the clonality status of infected individuals as the output of final states q1, q2 and q3. Transitions are described by the function δ, which specifies exactly one next state for each possible combination of state and input symbol. The rows in the transition table indicate the states Q, the columns the input symbols, and the table entries the transition function δ. We indicated the accepting state with an asterisk in the figures. F = q1, q2, q3 indicates the final states, which consist of a set of states Q.

The final state of q1 represents an early stage of clonal expansion in which clone sizes does not exceed the threshold of 512 infected cells. AC patients with PVL > 4% and the SM ATL patients with low PVL terminated in this state. The DFA of samples of clinically progressed patients with SM and chronic subtypes with maximum clone sizes of 2048 infected cells terminated at the final state of q2. The final state of q3 included samples of the SM, chronic and acute subtypes with clone size > 2048. Acute samples, which represent the final stage of ATL progression, were observed only in q3. VB clones were observed only in the samples whose DFA terminated at q3. In the current study, c4 (VB clone) was observed only once in each analyzed sample. Since, presence of more than one VB clone is theoretically possible, we put a loop on the q3 final state of our final DFA machine. In the case of observing such a sample, the clonality will be defined as a largely expanded oligoclonal pattern with q3 final state.

We conducted a cross-sectional analysis of HTLV-1–infected individuals with a broad range of PVLs, representing different progression states of disease. Although analyzing the same individuals over time is of great importance, obtaining these kinds of samples is difficult and needs to be addressed in future studies. However, having access to the JSPFAD biomaterials bank allowed us to obtain two longitudinal (2 years apart) samples from the same individual (F12 and F16). By analyzing these samples, we could directly examine the hypotheses that samples with a higher final state have a higher chance of disease progression. At the first time point (F12) the patient was diagnosed with chronic ATL and had a PVL of 83.81% and major clone size of 5377. At the second time point (F16), the patient had progressed to the acute stage and had a PVL of 51.42% and clone size of 4909. The PVL and size of the major clone at the second time point were presumably decreased because of therapy. However, the major clone with integration site of Chr9:123682855 (+) remained stable and dominant over the 2-year period. The DFA for both time points terminated in q3. Thus, it appears that reaching the final state of q3 is a factor that can be used as a risk indicator. Because the final state of this patient was already q3 at the first time point, progression to the acute stage was predicted by our DFA. As further validation of our DFA, the other patients with acute ATL also had DFA that terminated at q3, and thus we expected these patients to have a poor prognosis. Subsequently, we confirmed the poor prognosis of these patients (F13, F14, F15 and F16) by checking their clinical follow-up data, which showed that they had all died of the disease. However, AC patients (F1, F2 and F3) and patients with SM ATL with low PVL (F4) who showed the final state of q1 in the DFA remained clinically stable without disease progression in two years.

The data suggest that our final proposed machine (M) not only describes the clonality status of patients at single time points within a cross-sectional analysis but also opens the door for future analyses of longitudinal samples for predictive purposes. The predictive ability of this model with larger numbers of samples from the same individuals over time still needs to be examined.

We believe our model is an appropriate empirical model for this system because it uses real biological data without theoretical assumptions as well as the fewest number of variables and the simplest set of relationships to explain the clonality status of samples. This model has the potential to provide insight into clonal expansion of ATL, but we are still far from understanding exactly when, where and at which step clonal expansion and transformation occur and how they can be controlled. However, our multidisciplinary strategy for translating the data of clonal expansion into the computable language of mathematics via modeling opens new avenues to approach these relevant questions in future studies.

Currently, ATL patients are categorized into different subtypes of disease progression based on clinical manifestations [46]. These standard clinical criteria for diagnosis mainly include organ involvement, leukemic manifestation, and levels of lactate dehydrogenase and calcium [58]. However, molecular features that represent the disease status remain to be characterized. Considering that in clinical practice distinct therapeutic strategies are used for the treatment of different subtypes of ATL, accurate subtype classification is of great importance. Thus, there is demand for more robust classification of ATL subtypes mediated by a genomic feature, such as HTLV-1 clonal composition. This kind of analysis would be also helpful in clinical decision-making, such as monitoring the outcome of therapeutic interventions, based on analysis of the clonality status of patients before and after therapy [59]. In this respect, constructing an empirical model of clonal expansion would be one of the primary steps towards developing a powerful software tool for automated analysis and interpretation of individual clonality, which holds great promise for molecular diagnostics and personalized therapeutic interventions.

## Conclusions

We used HTLV-1 integration sites as a stable fingerprint to identify infected cells and accurately monitor their clonal expansion. We isolated large numbers of integration sites and quantified the clone sizes of eighteen clinical samples by our high throughput and validated methodology. We defined a threshold system that categorizes the size of clones into discrete groups based on the number of infected cells in each clone. We could quantify polyclonal, oligoclonal and monoclonal patterns using this categorization. We found that harboring larger clones was strongly associated with the progression of a patient to the more aggressive type of ATL, whereas smaller clones were observed across all samples and had little impact on progression. All samples with low PVLs (<10%) had smaller clones, however those with higher PVL had both smaller clones and one or two dominant larger clones. For the first time, we suggested DFA as a formalism that can represent sequential order of clones. We found that our DFA accurately reflect the true patterns of clonal expansion for each sample. Analyzing a large cohort of clinical samples from the same patients over time with the appropriate formal models will provide new insights into the clonal expansion of ATL and will allow for possible clinical applications of clonality in molecular diagnostics and predictions of prognosis.

## Additional files

Additional file 1: Table S1. Five largest clones and their integration-site positions for each sample. **Figure S1.** Overview of the library preparation for sequencing and data analysis. We used a specific pipeline to isolate integration sites from the raw NGS data. In the case of Read-1, the first 5 bp were trimmed, the next 5 bp were used to de-multiplex indexed samples, the 23 bp corresponding to the LTR primer were removed, the next 27 bp were subjected to a BLAST search against the long terminal repeat (LTR) reference sequence, and the remaining 40 bp were subjected to a BLAST search against an HTLV-1 reference sequence. Reads confirmed to be from HTLV-1 were removed, and the remaining reads were considered to be human. Using Bowtie, we then aligned those reads to the human genome (hg19). Subsequently, we retrieved data from Read-2 (tag information) to measure the clone sizes. The final output included information about the integration sites and clone sizes and was input as a string of information into our model. Finally we constructed DFA machines for each analyzed sample. **Figure S2.** Distribution of PVLs across the analyzed samples. The samples are displayed in descending order based on their largest clone size. (F18, F17, F1, F4, F2, F3, F10, F5, F6, F7, F9, F8, F15, F14, F13, F11, F16 and F12) on X axis. The corresponding PVLs of each sample are shown on Y axis. The PVLs and the size of largest clones had a correlation of R2 = 0.785. **Figure S3.** Clone size distribution of the five largest clones of each sample. The samples are displayed in ascending order based on their largest clones. Three main patterns, polyclonal, oligoclonal and monoclonal, were observed and categorized. The polyclonal pattern is divided (blue dashed line) into samples with PVL ≤ 4% and PVL > 4%. (PDF 386 kb)

## Abbreviations

ATL: Adult T-cell leukemia
HTLV-1: Human T-cell leukemia virus type-1
PVL: Proviral load
AC: Asymptomatic carrier
SM: Smoldering
JSPFAD: Joint Study on Predisposing Factors of ATL Development
NGS: Next generation sequencing
DFA: Deterministic finite state automata
