# Supplementary material for: Multidisciplinary insight into clonal expansion of HTLV-1–infected cells in adult T-cell leukemia via modeling by deterministic finite automata coupled with high-throughput sequencing
Source: BMC Med Genomics. 2017 Jan 31;10:4. doi: 10.1186/s12920-016-0241-2 (PMC5282739; doi:10.1186/s12920-016-0241-2)
Supplement: Additional file 1: Table S1. — Five largest clones and their integration-site positions for each sample. Figure S1. Overview of the library preparation for sequencing and data analysis. We used a specific pipeline to isolate integration sites from the raw NGS data. In the case of Read-1, the first 5 bp were trimmed, the next 5 bp were used to de-multiplex indexed samples, the 23 bp corresponding to the LTR primer were removed, the next 27 bp were subjected to a BLAST search against the long terminal repeat (LTR) reference sequence, and the remaining 40 bp were subjected to a BLAST search against an HTLV-1 reference sequence. Reads confirmed to be from HTLV-1 were removed, and the remaining reads were considered to be human. Using Bowtie, we then aligned those reads to the human genome (hg19). Subsequently, we retrieved data from Read-2 (tag information) to measure the clone sizes. The final output included information about the integration sites and clone sizes and was input as a string of information into our model. Finally we constructed DFA machines for each analyzed sample. Figure S2. Distribution of PVLs across the analyzed samples. The samples are displayed in descending order based on their largest clone size. (F18, F17, F1, F4, F2, F3, F10, F5, F6, F7, F9, F8, F15, F14, F13, F11, F16 and F12) on X axis. The corresponding PVLs of each sample are shown on Y axis. The PVLs and the size of largest clones had a correlation of R2 = 0.785. Figure S3. Clone size distribution of the five largest clones of each sample. The samples are displayed in ascending order based on their largest clones. Three main patterns, polyclonal, oligoclonal and monoclonal, were observed and categorized. The polyclonal pattern is divided (blue dashed line) into samples with PVL ≤ 4% and PVL > 4%. (PDF 386 kb) [file 12920_2016_241_MOESM1_ESM.pdf]

Additional file 1

Multidisciplinary insight into clonal expansion of HTLV-1–infected cells in adult T-cell leukemia via modeling by deterministic finite automata coupled with high-throughput sequencing

Amir Farmanbar[1, 2]*, Sanaz Firouzi[1]* §, Sung-Joon Park[2], Kenta Nakai[1, 2], Kaoru Uchimaru[1, 3], Toshiki Watanabe[4] §

Table S1 Five largest clones and their integration-site positions for each sample.

	chromosome	strand	position	clone size		chromosome	strand	position	clone size
F1	chr11	+	41829351	310	F2	chr20	+	24316441	357
	chr11	-	37042531	252		chr7	+	95400426	147
	chr7	-	121751209	65		chr13	+	114205746	58
	chr13	+	69268500	58		chr13	+	22347864	43
	chr18	-	46701047	55		chr9	+	25456529	35
F3	chr18	+	49381817	388	F4	chr11	+	21370963	314
	chr13	+	53440917	287		chr11	+	134725790	118
	chr8	-	15979761	206		chr1	+	193637249	50
	chr11	+	33508993	206		chr10	+	117279473	38
	chr7	+	66773811	167		chr2	+	99892988	35
F5	chr4	-	563510	1427	F6	chr8	+	36185116	1446
	chr20	+	58007412	552		chr19	+	36832671	1088
	chr5	+	62579400	263		chr4	-	123809335	67
	chr6	+	133958164	161		chr3	+	78371460	24
	chr3	-	126392249	60		chr13	-	57924441	15
F7	chr2	-	52008948	1904	F8	chr4	+	77458865	2055
	chr2	+	30533641	1690		chr18	+	55830356	1293
	chr11	-	107403005	317		chr3	-	178394075	47
	chr8	+	15975180	205		chr17	-	8554132	46
	chr1	-	19452173	20		chr3	+	178394114	30
F9	chr9	+	122319707	2029	F10	chr1	-	118064268	736
	chr10	+	51564266	361		chr10	-	38237441	725
	chr11	-	24035362	69		chr2	+	175498638	131
	chr6	-	116750624	64		chrX	+	109859795	70
	chr15	-	82540883	52		chr6	+	84338087	56
F11	chr22	+	21801925	4883	F12	chr9	+	123682855	5377
	chr15	-	69983466	25		chr2	-	74467951	14
	chr4	-	172761457	24		chr12	+	71477801	8
	chr1	-	99992309	12		chr16	-	69802148	7
	chr4	-	48519702	11		chr14	+	90033247	3
F13	chr11	+	64086567	3721	F14	chr9	-	22270886	2848
	chr4	-	190566047	871		chr12	+	34513865	158
	chr6	-	50024650	774		chr7	+	146900801	138
	chr8	-	85288622	769		chr18	-	45635534	121
	chr11	+	15838900	475		chr4	+	69213533	117
F15	chr7	-	9408533	2634	F16	chr9	+	123682855	4909
	chr14	+	79681747	13		chr2	-	74467951	17
	chr9	+	128547479	9		chr1	-	163253696	3
	chr16	+	79518819	4		chr4	-	190566045	2
	chr3	-	52008197	3		chr12	-	40017236	2
F17	chr3	-	70309315	112	F18	chr2	-	74467951	77
	chr15	+	48948061	54		chr4	+	14403984	29
	chr13	-	35865120	47		chr7	+	117768673	28
	chr6	-	144903319	42		chr12	+	130675957	27
	chr3	+	56866376	39		chr6	+	20407115	26

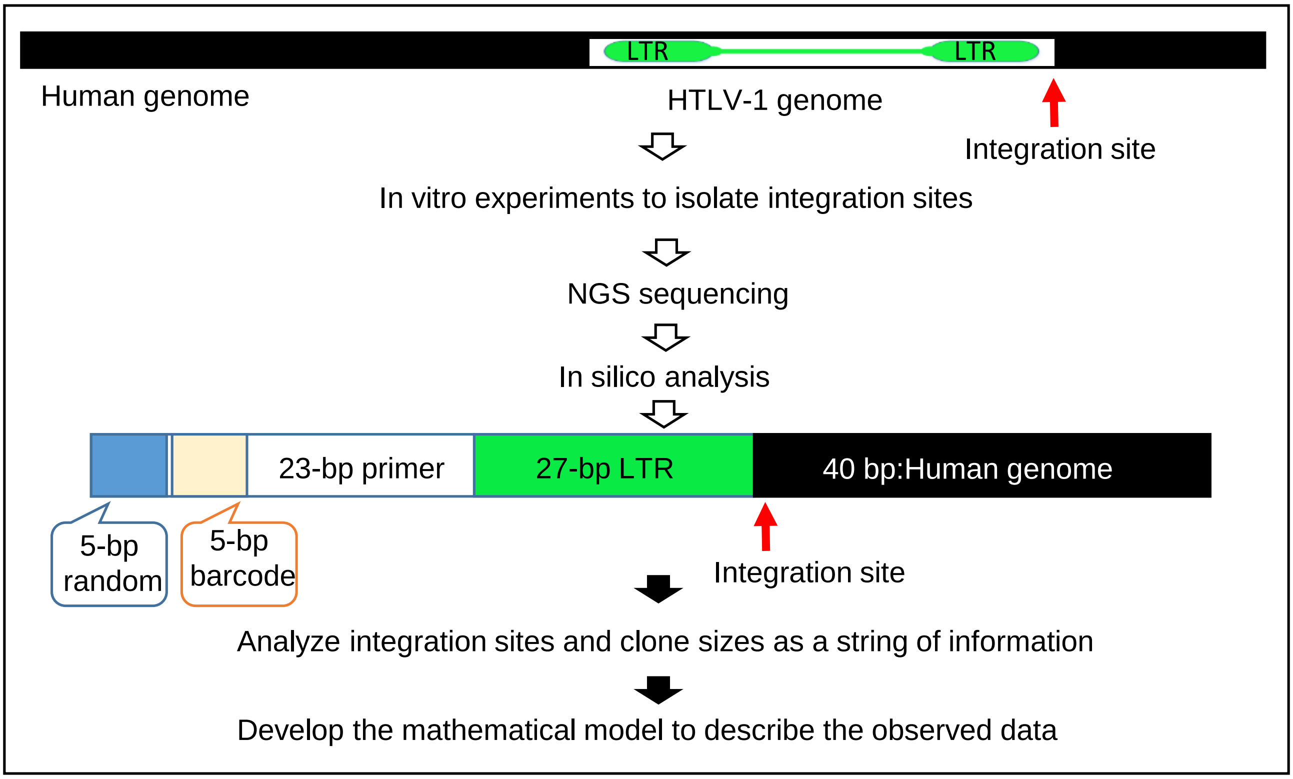

Figure S1
Overview of the library preparation for sequencing and data analysis. Genomic DNA isolated from PBMCs of clinical samples underwent *in vitro* preparation steps to generate fragments for sequencing. We used a specific pipeline to isolate integration sites from the raw NGS data. In the case of Read-1, the first 5 bp were trimmed, the next 5 bp were used to de-multiplex indexed samples, the 23 bp corresponding to the LTR primer were removed, the next 27 bp were subjected to a BLAST search against the long terminal repeat (LTR) reference sequence, and the remaining 40 bp were subjected to a BLAST search against an HTLV-1 reference sequence. Reads confirmed to be from HTLV-1 were removed, and the remaining reads were considered to be human. Using Bowtie, we then aligned those reads to the human genome (hg19). Subsequently, we retrieved data from Read-2 (tag information) to measure the clone sizes. The final output included information about the integration sites and clone sizes and was input as a string of information into our model. Finally we constructed DFA machines for each analyzed sample.

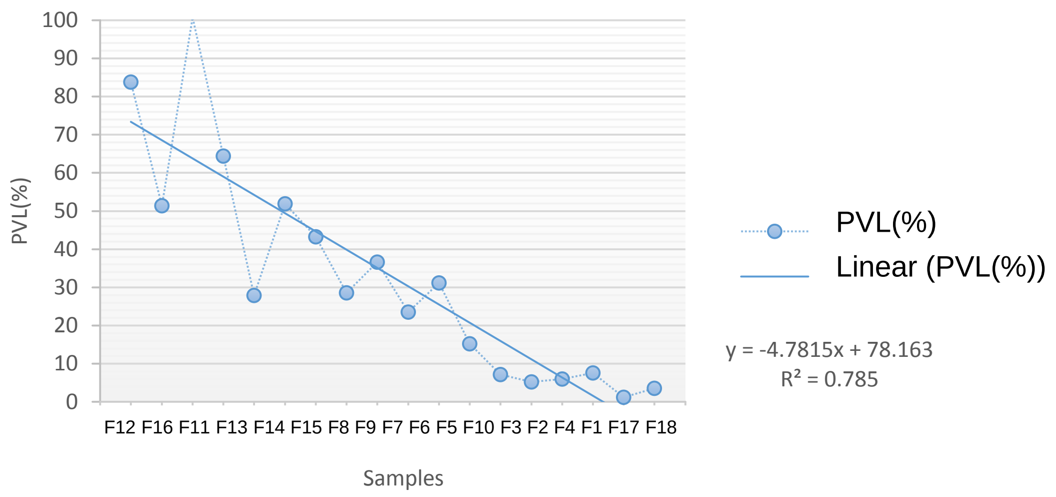

Figure S2

Distribution of PVLs across the analyzed samples. The samples are displayed in descending order based on their largest clone size. (F18, F17, F1, F4, F2, F3, F10, F5, F6, F7, F9, F8, F15, F14, F13, F11, F16 and F12) on X axis. The corresponding PVLs of each sample are shown on Y axis. The PVLs and the size of largest clones had a correlation of R2=0.785.

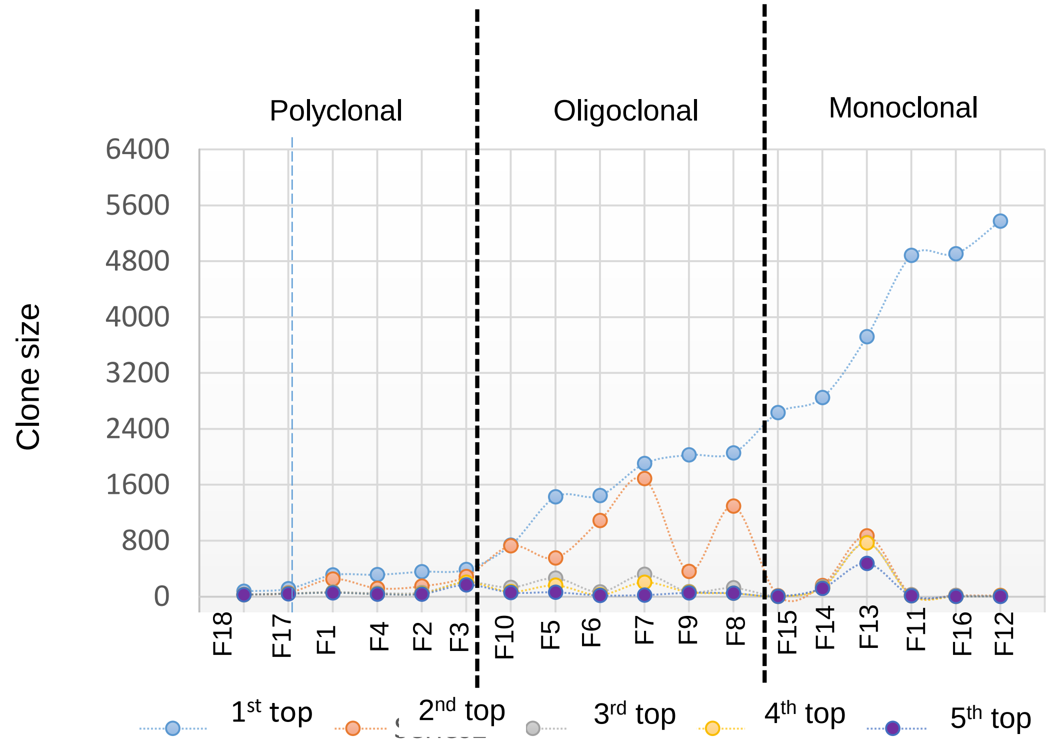

Figure S3

Clone size distribution of the five largest clones of each sample. The samples are displayed in ascending order based on their largest clones. Three main patterns, polyclonal, oligoclonal and monoclonal, were observed and categorized. The polyclonal pattern is divided (blue dashed line) into samples with PVL ≤ 4% and PVL > 4%.
